# Perspective on the Development and Validation of Ab Reagents to Fish Immune Proteins for the Correct Assessment of Immune Function

**DOI:** 10.3389/fimmu.2018.02957

**Published:** 2018-12-13

**Authors:** Brian Dixon, Daniel R. Barreda, J. Oriol Sunyer

**Affiliations:** ^1^Department of Biology, University of Waterloo, Waterloo, ON, Canada; ^2^Department of Biological Sciences, and Department of Agricultural, Food and Nutritional Science, University of Alberta, Edmonton, AB, Canada; ^3^Department of Pathobiology, School of Veterinary Medicine, University of Pennsylvania, Philadelphia, PA, United States

**Keywords:** immunology, teleost fish, antibodies, assays, validation, reagents

## Abstract

Understanding of immune function in humans and model organisms, such as mice, has advanced in the last few decades because of technological breakthroughs and availability of reagents. While novel genomic technologies have helped to increase knowledge of many aspects of immunology, most developments in immunology have occurred because of the availability of antibodies to identify and sort different cell types, as well as to identify and quantify the protein products of cells. Unfortunately, many studies performed in fish make use of poorly characterized antibody reagents that may affect the conclusions of those studies. In light of this, we would like to offer some insight and discussion points based on our research experience on the strategies and techniques that are required for proper validation of antibody reagents to fish immune molecules. Our main goal is to encourage a much needed discussion in our field to foster the use of correctly validated reagents that enable the study of fish immune function.

## Introduction

Genomic technology is advancing rapidly because nucleic acids can be amplified and, thus, methods for inexpensive large-scale sequencing have been developed. In non-classical model species, like those comparative immunologists use, genomics is a key first step because specific reagents to one particular gene or protein are not usually available. While microarrays or RNAseq can determine mRNA concentrations of all genes expressed in a cell or tissue, in some cases this may not necessarily reflect transcription rates, as mRNA degradation by RNAses plays a role in determining mRNA, especially for certain genes like cytokines and chemokines with degradation motifs in their 3′ untranslated regions (1). Thus, tools to measure proteins and cells are required to understand immune function.

## Transcripts vs. Proteins

Transcript concentrations that nucleic acid-based assays detect may directly relate to protein concentrations, but for many genes they may not, and will certainly not reflect concentrations of active protein. This is especially true for many of the genes that immunologists are interested in. For example, receptors must be translated into protein through specific channels into the endoplasmic reticulum (ER), perhaps glycosylated and folded by chaperones, then transported through the Golgi to the surface. These steps all depend on the dynamics and rates of action of several enzymes and transport molecules that cannot be assessed by examining mRNA concentrations. Cytokines must also be translated into the ER and transported to the cell surface for secretion. Many are secreted in inactive forms (glycosylated or as precursors that need to be cleaved i.e., IL1β). These processes all have dynamics that RNA based assays cannot capture. In addition, receptors or high affinity binding receptor subunits (e.g., IL2Rα) must be upregulated in a coordinated fashion for full function and, in some cases, decoy receptors bind the ligands to prevent their function and/or decoy ligands compete for the active receptor [e.g., as seen for IL1β, see (2, 3)]. Often these molecules are expressed in a different cell type or tissue than the one studied by RNA based assays, which can be addressed through inclusion of additional experimental steps but should be considered carefully when designing experiments. Another example of mismatch between mRNA concentrations and functional capacity that is very familiar to immunologists is complement. Complement component C3 must be cleaved to be activated and simply measuring C3 mRNA cannot give a clear picture of the level of complement activity at any given point in time, especially since for C3, like many teleost immune genes, there are multiple isoforms present in the genome, all of which may have different dynamics and roles (4).

For those of us that have chosen to work in mixed cell populations or at the tissue level, it is important to consider that many target genes are differentially expressed among cells (types and developmental stages) and may further exert different functions depending on which cell expresses them [e.g., consider TLRs; (5)]. Large populations of immune cells also migrate actively through various tissues further complicating assessments when single tissues are examined. Cell-cell interactions are also critical for immune functions. Even as single cell transcriptomic technologies become increasingly accessible, these approaches are unlikely to capture cell-cell interactions that are critical for immune function. Coupled to the challenge of working with outbred populations of animals, this can introduce significant variability in the resulting datasets and make it much harder to assess functional implications. RNA based assays on a whole tissue will capture and average mRNA expression in interacting cells, along with several other cell types, making understanding how each reacts to the other impossible. Also, not all transcripts make protein: MicroRNA (miRNA) regulation of many genes, including immune genes of fish [e.g., see (6, 7)], means that a measurement of the total amount of mRNA present does not accurately reflect the amount of protein that will ultimately be produced. Most critically, as new opportunities arise to understand adaptive mechanisms in fish, one common scenario continues to be seen: changes in the transcription of the IgM, IgT/IgZ, and IgD genes are being used to evaluate antibody responses. While these genes may be upregulated during immune responses, such an increase is not indicative of a specific antibody response because one cannot identify an immunoglobulin with specificity for the antigen in question from mRNA expression. Secondly, while in mammals there might be an isotype switch indicative of a specific response (e.g., from IgM to IgG), detectable using mRNA expression data, this is not possible for species like teleost fish where there is no isotype switching. Primers used in many studies to amplify fish immunoglobulin transcripts, also do not distinguish between sterile and productive transcripts. Finally, in humans, not all circulating immunoglobulins are active: immunoglobulins bearing sugar moieties with terminal sialic residues are anti-inflammatory and the structure of these carbohydrates' changes during immune responses to forms with terminal N-acetylglucosamine residues that have much more bioactivity (8). This is likely to be similar in other species; for example, rainbow trout IgM heavy chains have five potential N-linked glycosylation sites (9) while IgT isoforms have at least two (10, 11).

Thus, caution is needed in forming conclusions solely from RNA based studies without complementing them with functional data at the protein, cellular, and/or organismal levels. RNA based assays may be useful as first experiments to focus future work on the correct cells and proteins and can provide important context as we dissect mechanisms of immune function but cannot be the final experiment used to make conclusions about immune functions, processes, and responses.

## Kinetics

Exhaustive analysis of cellular or molecular events occurring during a typical immune response is unrealistic because of funding and time constraints. However, on the other extreme, we continue to see costly decisions being made in both academia and industry based on evaluations of individual or very limited time points. Regardless of the analytical depth (molecular or cellular), no single time point can provide sufficient functional context for the effectiveness of an immune response and, thus, added emphasis should be placed on kinetics rather than the robustness of select parameters at a single time point. The number of relevant time points and the level of depth in which they should be evaluated will vary depending on the biological question. However, effects on immune competence (e.g., due to infection, environment, diet, therapy) need to consider changes to the efficiency of induction of immune mechanisms, their absolute concentrations, and whether a timely return to homeostasis has been achieved (e.g., required to minimize unnecessary tissue damage and unproductive use of metabolic energy resources). For all of these, it is paramount that molecular datasets are complemented by evaluation of functional responses (e.g., cell function, pathology, host performance).

## Developing Antibodies to Assess Fish Immune Function

In addition to using transcript concentrations as proxy of functionality, fish immunologists need antibodies against immune molecules and cells in order to understand specifically how the fish immune system operates. Unfortunately, developing and validating such tools is slow, painstaking work, regardless of the animal model used. Most importantly, the correct standardization and validation of these antibodies is fundamental for comparison of results among different labs. Below are some key areas that require special attention during the production and validation of polyclonal (pAb) and monoclonal (mAb) antibodies to fish molecules.

### Developing Antibody Targets

In most instances, the antibody target cannot be purified in its native form, and thus, recombinant proteins must be produced. Soluble proteins expressed in prokaryotic expression systems usually require proper refolding to acquire a more accurate conformational structure. Thus, a refolding step has proven critical for some of our prokaryotically-produced antigens in order to induce antibody responses that will recognize the native fish molecule (12). This is especially true in immune assays in which these reagents are required to recognize the native molecule (i.e., ELISA, flow cytometry). Antibodies may also be developed to proteins that are expressed in mammalian cells and then reintroduced into the host, for example, expression in rat cells and injection into rats. Antibodies may also be made to peptides as long as care is taken to ensure the protein fragment is expressed on the outside of the folded protein. In our experience, antigens produced in eukaryotic expression systems are better at inducing antibodies that recognize the native fish molecule compared to antigens made in prokaryotic expression systems. Antibodies developed to mammalian molecules that have a high degree of sequence identity to the equivalent fish proteins can sometimes be used, but must be validated very carefully using some of the methods suggested below to ensure that they do indeed bind to the correct target.

### Immunization and Adjuvants

In our experience, the use of rabbits for the production of pAbs against fish molecules is not ideal. Rabbit antibodies appear to naturally recognize, or non-specifically bind to a significant percentage of fish leukocytes and proteins, producing false positive results, and high background, perhaps due to cross priming. This is particularly critical for flow cytometry, in which the real reactivity is confounded by the cross-reactive/non-specific binding capacity of these rabbit Igs. Antibodies produced in guinea pigs, rats, mice, and chickens do not usually present this problem. For mAb production, the use of more than one species (i.e., mice and rats) provides alternative choices for secondary reagents when the experiment involves several mAbs to different (e.g., flow cytometry) or the same (e.g., ELISA) antigen. The choice of adjuvant may amplify the titers of non-specific or cross-reactive Abs, especially in rabbits, and thus, it is worth exploring which adjuvant (e.g., oil-based vs. non-oil-based) works best for your antigen. In many cases, it is incorrect to assume that the pre-immune serum is a good control, since those animals have not been exposed to adjuvant. Serum from animals injected with adjuvant alone might be more appropriate in some cases. Control serum should be subjected to the same purification steps as immune serum in order to ensure that background reactivity that remains is consistent.

### Characterization and Validation

For pAbs, it is crucial to use antibodies affinity purified against the antigen (i.e., using an affinity column) after protein A or a similar purifying agent has been used to obtain the pure Ig fraction. It is also helpful to affinity purify polyclonal serum against recombinant proteins produced in two different systems (i.e., bacterial and eukaryotic) to minimize potential problems of non-specificity or cross-reactivity.

Without a doubt, the most critical aspect in the development of pAb or mAbs, is the strategy used to validate such reagents. Below we describe some of the most critical steps we have used for the correct validation of Ab reactivity:

#### General Validation Strategies

Since recombinant antigen is not usually produced in fish cell lines, it is likely that critical antigenic sites are lost when proteins expressed in prokaryotic or eukaryotic expression systems do not fold correctly. Therefore, it is mandatory to test whether the Abs induced by those recombinant antigens recognize the native fish molecule, as recognition of recombinant antigen by these Abs does not necessarily translate into recognition of the native molecule. In our view, one of the best strategies is to assess whether these Abs recognize the native antigen when recombinantly expressed on a fish cell line of the same or similar species used for your experiments. For example, recently produced mAbs to rainbow trout CD4-1 and CD4-2 molecules were validated by showing that these mAbs could recognize those molecules transiently expressed on a rainbow trout cell line (13). Moreover, recognition of antigens expressed in a cell line should be checked by at least two different techniques, including flow cytometry and western. Another effective strategy to validate the correct reactivity of the Abs is to immunoprecipitate or column purify the native antigen from fish serum or leukocytes. This strategy is most valuable when combined with sequencing of the purified protein to confirm its identity, but requires that antigen is produced in significant levels and that it displays sufficient stability.

#### Validation Strategies of Abs to CDs

When producing Abs to CDs to detect specific fish leukocyte populations, additional validation steps to those described in section General validation strategies are required, to confirm that the Abs recognize the expected leukocyte subset. Thus, until more CD markers for fish cells become available, one might sort the cell subset/s recognized by the Abs (ideally by FACS) and perform RT-PCR on the sorted cells to confirm the expression of target cell-type transcripts for the target cells. Equally important, is measurement for transcripts uniquely expressed by other leukocyte subsets to rule out cross-reaction or non-specific recognition of surface molecules of unrelated leukocyte populations. For instance, if the newly produced mAb recognizes CD4, then sort the cells recognized by this mAb and check not only for the expression of CD4 transcripts but also for the expression of molecules typically expressed by other cell leukocytes (i.e., CD8 T cells, immunoglobulin, NK cell receptors, etc.). If the sorted leukocyte population only expresses CD4 T cell transcripts, then it indicates that the Ab is highly specific. The possibility exists however that we may find unexpected fish cell populations expressing CDs found only in certain leukocyte subsets in mammals, in which case further characterization of those potentially new cell subsets is needed to validate the Abs. Thus, it is important to keep an open mind and not assume that fish leukocyte subsets will express the same CDs expressed by their mammalian counterparts.

#### Validation Strategies of Abs to Fish Cytokines

In addition to the validation steps described in section General validation strategies, there are some peculiarities related to the validation of Abs raised against fish cytokines. To date, only a few antibodies to fish cytokines have been reported, and while they work very well on immunoblotting assays, further validation is required before any antibody can be used for more quantitative assays or to detect native molecules. It has been very difficult to develop sandwich ELISAs to measure specific cytokine concentrations in fish fluids, and/or to detect fish cytokines intracellularly. For instance, in the last 5 years two of us (Dixon and Sunyer labs) have attempted to produce mAbs and pAbs to over 15 different fish cytokines, and after performing all of the pertinent validation steps, Sunyer's lab could only validate with a high degree of confidence Abs to one out of eight target cytokines in tissues and fish fluids (Sunyer's personal communication) while Dixon's lab is confident that one target cytokine of seven can be detected. In some cases, the produced Abs did recognize the recombinant cytokine, but could not recognize the native fish cytokine (Dixon's and Sunyer's personal communication). In other instances, the Abs could recognize both the recombinant antigen and the native cytokine when expressed on a trout cell line, however, we were unable to detect the cytokine in any fish fluid, or intracellularly by means of ELISA, western blotting, or flow cytometry. Thus, it is apparent that some of these fish cytokines may be expressed at very low concentrations (i.e., Dixon's lab successful ELISA detects IL-1β at concentrations below 100 pg/mL in serum) or require the proper stimulation of the cell type producing them, in order to be detected. For example, detection of IL-10-producing B cells in mammals requires re-stimulation of these cells *ex-vivo* in order detect intracellular IL-10 (14). There may also be factors in the fish tissues that inhibit ELISA reactions as Dixon's group has shown that chloroform extraction enhances detection by ELISA and shows expression profiles that match qPCR and Western data in some cases—for example IL-1β in serum. Moreover, the detection of intracellular cytokines in mammals typically requires the use of brefeldin or monensin, both protein transport inhibitors, to enhance the accumulation of intracellular cytokine (15). However, the application of all of the above measures to enhance the intracellular detection of cytokines by flow cytometry is not a guarantee of success. For example, while Sunyer's lab has produced mAbs and pAbs to trout IL-10 and have successfully developed a sandwich ELISA that detects soluble native trout IL-10 (unpublished results), they have not been able to consistently assess intracellular IL-10 expression in trout lymphocytes. They have tried multiple combinations of *in vitro* re-stimulation cocktails and protocols, different kinetics, different concentrations of brefeldin and monensin, different cell permeabilization protocols, and different combinations of all of the above without success. However, IL-10 could be detected in the cell supernatants in many cases, which indicates a failure to detect the intracellular IL-10 specifically. Alternatively, Sunyer's lab has developed immunohistochemistry protocols to identify the cell types producing IL-10 (Sunyer's personal communication). Dixon's group can detect IL-1β in serum, cell culture supernatants, and cell extracts, although Western blots show bands of odd sizes that are difficult to reconcile in the latter case. These may or may not be background band because the true size of the native protein *in vivo* is unknown and is unlikely to match the predicted size based on amino acid sequences. Native IL-1β, may not even be a single size because like IL-10 and many other cytokines, it can be glycosylated at one or more sites *in vivo*. This effects stability and biological activity in ways that are unclear even in mammals (16), but can inhibit function (17) or enhance receptor binding (18). This glycosylation may indeed cause the problems in intracellular staining noted by Sunyer's group as the carbohydrates may block or mask the epitopes recognized by the antibodies and glycosylation may differ between extracellular and intracellular compartments. Thus, an understanding of the basic biology of each cytokine is absolutely required before one can be confident in antibody staining and quantification techniques. Unfortunately, that will take time and effort—for example Dixon's lab is now deglycosylating extracts and is sequencing bands detected by his antisera that are not the “predicted” size to see if they actually represent alternative forms of the target cytokine.

## Conclusions

A true understanding of immune system function in fish absolutely requires that we do not simply ascribe function based on transcript profiles only, but that we develop antibodies and, most importantly, validate those reagents very carefully based on a detailed understanding of the basic biology of the target molecules and cells specifically in fish. We then need to share those reagents widely and wisely to further validate them, so that we can all advance knowledge together to not only improve fish immunology academically, but also partner and profit with the industries that depend on the knowledge we produce.

## Author Contributions

All authors listed have made a substantial, direct and intellectual contribution to the work, and approved it for publication.

### Conflict of Interest Statement

The authors declare that the research was conducted in the absence of any commercial or financial relationships that could be construed as a potential conflict of interest.
